# Gefitinib-loaded DSPE-PEG2000 nanomicelles with CD133 aptamers target lung cancer stem cells

**DOI:** 10.1186/s12957-017-1230-4

**Published:** 2017-08-30

**Authors:** Xiaolong Huang, Jingsong Huang, Dewen Leng, Shuo Yang, Qi Yao, Jin Sun, Jun Hu

**Affiliations:** 1 Department of Respiratory Medicine, Wuhan NO. 1 Hospital, 215 Zhongshan Street, Wuhan, 430022 China; 2 Laboratory Medicine, Third Hubei Provincial People’s Hospital, Zhongshan Street, Wuhan, 430022 China; 3 Department of Critical Care Medicine, Wuhan NO. 1 Hospital, 215 Zhongshan Street, Wuhan, 430022 China; 4 Department of Otolaryngology, Wuhan NO. 1 Hospital, 215 Zhongshan Street, Wuhan, 430022 China; 5 Department of Pharmacy, Second Military Medical University, 325 Guohe Road, Shanghai, 200433 China; 6 Department of Physical examination, Wuhan Hospital for Occupational Disease Prevention and Treatment, 18-20 Jianghanbei Road, Wuhan, 430016 China

**Keywords:** Nanomicelles, Lung cancer, Gefitinib, Cancer stem cells, CD133

## Abstract

**Background:**

Lung cancer stem cells (CSCs) are considered to be the seed of lung cancer, and CD133 is a marker of lung CSCs. Here, we developed gefitinib-loaded poly(ethylene glycol) 2000-distearoylphosphatidylethanolamine nanomicelles with CD133 aptamers (M-Gef-CD133) to eliminate CD133^+^ lung CSCs.

**Methods:**

M-Gef-CD133 was prepared using a lipid-film-based approach. The targeting and activity of M-Gef-CD133 towards lung CSCs were evaluated.

**Results:**

M-Gef-CD133 were small (25 nm) and showed enhanced cytotoxic effect towards CD133^+^ lung CSCs compared with non-targeted M-Gef and gefitinib. Notably, M-Gef-CD133 could significantly reduce tumor sphere formation and the percentage of CD133^+^ lung CSCs, indicating that it possesses selective toxicity against CD133^+^ lung CSCs.

**Conclusions:**

The interaction of CD133 aptamers and CD133 shows promise in the delivery of gefitinib to CD133^+^ lung CSCs, and M-Gef-CD133 represents a promising treatment to target lung CSCs.

## Background

Lung cancer is a leading cause of cancer death and predicted to be the leading cause of cancer death in 2017 in the USA [1]. In 2015, lung cancer was the leading cause of cancer deaths in men 75 years or older in China [2]. Thus, it is urgent to propose effective treatment for lung cancer. However, the lung cancer survival is hampered by its recurrence, multi-drug resistance, and metastasis [3, 4]. Lung cancer stem cells (CSCs) are considered to initiate lung cancer [5]. Some researchers hold the opinion that lung CSCs contribute to the recurrence and metastasis of lung cancer [6]. Thus, the elimination of lung CSCs could increase the therapeutic efficacy of drugs against lung cancer. The CD133 antigen is a putative CSCs marker in solid tumors including lung cancer [7–12]. CD133^+^ lung cancer cells have been shown to possess stronger potential than CD133^−^ lung cancer cells in self-renewal, proliferation, differentiation, and in vivo tumor formation in mice [11, 12].

Targeted nanoparticles with ligands such as aptamers or antibodies have attracted extensive attentions, since they could improve the targeting efficacy of chemotherapy drugs [13–15]. Although antibodies are widely applied in targeted nanoparticles, they have disadvantages such as strong immunogenicity [15]. Aptamers—oligonucleic acids—have advantages including low immunogenicity, low molecular weight, and easy production, making them ideal targeted ligands [16]. The aptamer A15 has been demonstrated to be a promising ligand for targeting CD133^+^ cells [17]. Given that CD133 is a marker of lung CSCs, we hypothesize that A15–CD133 interaction could mediate effective delivery of drugs to CD133-positive lung CSCs.

Nanomicelles are nanoparticles by self-assemblies of block copolymers [18, 19]. Poly(ethylene glycol) 2000-distearoylphosphatidylethanolamine (DSPE-PEG2000) nanomicelles are promising owing to their small size and superior penetration [20–22]. We hereby constructed gefitinib-loaded DSPE-PEG2000 nanomicelles with CD133 aptamers (M-Gef-CD133) to target lung CSCs. Gefitinib (Iressa), a tyrosine kinase inhibitor of epidermal growth factor receptor (EGFR) for treatment of non-small cell lung cancer (NSCLC) [23, 24], was used as a model drug in the study.

## Methods

### Reagents

1,2-Distearoyl-sn-glycero-3-phosphoethanolamine-*N*-[maleimide (polyethylene glycol)-2000] (DSPE-PEG2000-Mal), 1,2-distearoyl-sn-glycero-3-phosphoethanolamine-*N*-[methoxy(polyethylene glycol)-2000] (DSPE-PEG2000), and 1,2-dioleoyl-sn-glycero-3-phosphoethanolamine-*N*-carboxyfluorescein (CFPE) were bought from Avanti Polar Lipids (Alabaster, AL). Phycoerythrin-labeled CD133 antibodies and the CD133 MicroBead Kit were bought from Miltenyi Biotec (Auburn, CA). Gefitinib was bought from Dalian Meilun Biotech (Dalian, China). The primers and thiolated CD133 aptamer with a sequence of 5′-SH-CCCUCCUACAUAGGG-3′ were bought from Ruibo Co., Ltd. (Guangzhou, China). *N*-Hydroxysuccinimide (NHS), 1-ethyl-3-3-dimethylaminopropyl carbodiimide (EDC), epidermal growth factor (EGF), and basic fibroblast growth factor (bFGF) were bought from Sigma-Aldrich (St. Louis, MO). The StemPro® Accutase® Cell Dissociation Reagent, the Reverse Transcription System kit, TRIzol® Reagent, B27, and insulin-transferrin-selenium (ITS) were bought from Thermo Fisher Scientific Inc. All organic reagents (analytical grade) were provided by Sinopharm (Shanghai, China) unless otherwise stated.

### Cell culture

Human lung cancer cell lines A549 (ATCC® CCL185™, human epithelial lung cancer cells) and A431 (ATCC® CRL1555™, human epithelial epidermoid cancer cells) were provided by the American Type Culture Collection (Manassas, VA). The cells were cultured in RPMI 1640 medium with l-glutamine supplemented with 10% FBS (fetal bovine serum) and 0.1 mM MEM non-essential amino acids in a cell incubator at 37 °C with a humidified atmosphere containing 95% air/5% CO_2_.

### Real-time polymerase chain reaction (RT-PCR)

An RT-PCR assay was performed to examine the expression of CSC-related genes in lung cancer cells. Briefly, the first-strand complementary DNA was reverse transcribed with the Reverse Transcription System kit, after RNA was extracted with the TRIzol® Reagent. A Roche Light Cycler (Mannheim, Germany) performed the RT-PCR. The mRNA expression was expressed as 2^CΔΔCT^ [25].

### CD133 expression analyzed by flow cytometry

The collected cells were incubated with PE-CD133 antibodies (1 μg/mL) at 4 °C for 30 min, and the cells were analyzed for CD133 expression using flow cytometry (Becton Dickinson, San Jose, CA).

### Magnetic cell sorting

The CD133^+^ cells were isolated from the lung cancer cells with magnetic cell sorting (Miltenyi Biotec.). Briefly, CD133 microbeads were added to the collected cells. Uncombined microbeads were discarded after mixing the CD133 microbeads with the cells at 4 °C for 15 min. Then, the pellet was resuspended with 500 μL PBE (PBS with 5 mM EDTA and 0.5% BSA) and separated using a magnetic separation column. After being washed, the CD133^+^ cells attached to the column were collected. Finally, the CD133 expression of the cells was analyzed by flow cytometry.

### Tumor sphere-forming assays

Tumor sphere-forming assays are used to identify CSCs, based on their capacity to realize differentiation and self-renewal at the level of single cells [26]. Briefly, lung cancer cells (3000 cells/well) were cultured in ultra-low adherent six-well dishes (Corning, Tewksbury, MA). The cells were suspended in stem cell medium composed of DMEM/F12 (Dulbecco’s modified Eagle medium/Ham’s F-12 medium), with 1 × B27, 1 × ITS, 20 ng/mL EGF, and 20 ng/mL bFGF. The number of tumor spheres was counted after 7 days. For the formation of the second-passage tumor spheres, tumor spheres were dissociated by the StemPro® Accutase® Cell Dissociation Reagent and propagated.

### Development of gefitinib-loaded nanomicelles (M-Gef)

Gefitinib-loaded nanomicelles (M-Gef) were developed with the lipid-film-based approach. In brief, 2 mg gefitinib and 10 mg DSPE-PEG2000 were dissolved in 5 mL chloroform. Then, the solvent was evaporated to form a dry lipid film. Using 5 mL phosphate-buffered saline (PBS; pH 7.4), the dried film was hydrated. With filtration through 200-nm polycarbonate membranes (Nucleopore, Whatman), free gefitinib was removed from the nanomicelles. Fluorescent nanomicelles were fabricated with the addition of 1% CFPE (mass ratio) in the lipid.

The targeted nanomicelles—gefitinib-loaded nanomicelles with CD133 aptamers (M-Gef-CD133)—were fabricated by conjugating CD133 aptamers to M-Gef by a thiol-maleimide reaction [27, 28]. In brief, M-Gef was prepared as described above, but 10 mg DSPE-PEG2000 was replaced by 10 mg lipid mixture consisting of 2 mg DSPE-PEG2000-Mal and 8 mg DSPE-PEG2000. The resultant M-Gef was incubated with CD133 aptamers (0.1 mg) for 6 h under stirring. After ultrafiltration with PBS, the M-Gef-CD133 was resuspended in PBS for use.

The nanomicelles were designated as follows: M-Gef-CD133 (gefitinib-loaded nanomicelles with CD133 aptamers), M-Gef (gefitinib-loaded nanomicelles), M-CD133 (blank nanomicelles conjugated with CD133 aptamers), CFPE-M-Gef-CD133 (CFPE-labeled gefitinib-loaded nanomicelles conjugated with CD133 aptamers), and CFPE-M-Gef (CFPE-labeled gefitinib-loaded nanomicelles).

### The size, size distribution, zeta potential, and morphology of nanoparticles

With a Zetasizer Nano S (Malvern Instruments, UK), the size, size distribution, and zeta potential of nanomicelles were analyzed. The morphology of the nanomicelles were examined using Hitachi H-600 transmission electron microscopy (TEM, accelerating voltage of 200 kV).

### The encapsulation efficiency and loading of salinomycin in nanomicelles

After being vacuum dried, 1 mL nanomicelle solution was completely dissolved in 1 mL methanol for the analysis with high-performance liquid chromatography (HPLC; L-2000, Hitachi, Japan) equipped with a reverse phase C-18 column (Diamonsil, 5 μm, 250 mm × 4.5 mm). Chromatographic conditions (mobile phase: 0.02 M dipotassium hydrogen ortho phosphate/methanol (10/90, *v*/*v*), detection wavelength: 246 nm, column temperature: 30 °C, and flow rate: 1 mL/min). The encapsulation efficacy of gefitinib = the masses of encapsulated gefitinib/the mass of total added gefitinib × 100%. The drug loading of gefitinib = the masses of encapsulated gefitinib/the mass of gefitinib-loaded nanomicelles × 100%. The CFPE concentration of nanomicelles was measured using a CFPE calibration curve.

### In vitro release of gefitinib

The release of gefitinib in vitro was examined in phosphate buffer solution (PBS, pH 7.4) or PBS with 10% human plasma. Briefly, add 5 mL nanomicelle solution to the Spectra/Por^®^ dialysis membrane (MWCO 1000). After being introduced into a vial containing release medium, the sealed tube was incubated in a water bath under gentle stirring at 37 °C (100 rpm). Two-milliliter aliquots of the dialysate were replaced with 2-mL fresh medium at different time points. The amount of gefitinib in the dialysate was measured by HPLC as described above.

### Evaluation of in vitro cellular uptake of nanomicelles using flow cytometry

Lung cancer cells (5 × 10^5^ cells/well) were inoculated in 12-well plates overnight. The cells were then incubated with CFPE, CFPE-M-Gef, or CFPE-M-Gef-CD133 (0.5 μg/mL equivalent CFPE concentration) for 4 h. Finally, the cells were analyzed with flow cytometry.

### Evaluation of in vitro cellular uptake of gefitinib by HPLC

The intracellular uptake of gefitinib was measured by HPLC. Briefly, lung cancer cells (5 × 10^5^ cells/well) were inoculated in 12-well plates overnight. Then, the cells were treated with gefitinib, M-Gef, or M-Gef-CD133 (20 μg/mL equivalent gefitinib concentration) for 4 h. The cells were then washed thrice with PBS and collected by adding 0.4 mL methanol. After the cells were sonicated for 1 min, the cell lysate was centrifuged (10,000×*g* for 10 min), and the supernatant was collected. HPLC quantified the gefitinib content in the supernatant. The protein concentration of supernatant was determined by the BCA™ Protein Assay Reagent Kit (Pierce). The intracellular uptake of gefitinib = gefitinib concentration/protein concentration.

### Cell proliferation assays measured using CCK-8

The cytotoxic effect of the nanomicelles was examined with the CCK8 assay. Briefly, cells (1 × 10^4^ cells/well) were inoculated in 96-well plates overnight. Then, the cells were treated with various concentrations of drugs (0.15~3000 μg/mL) for 48 h, and cell proliferation was examined according to the protocol of the kit.

### The effect of nanomicelles on the CSC proportion of lung cancer cells

The effect of nanomicelles on the CSC proportion in lung cancer cells was examined by the tumor sphere formation and proportion of CD133^+^ cells. In brief, lung cancer cells (5 × 10^4^ cells/well) were inoculated in 12-well plates overnight. The cells were then treated with the nanomicelles or free gefitinib (5 μg/mL equivalent gefitinib concentration). After 24 h, the drugs were discarded, and the cells were incubated with fresh medium for 72 h. Then, the cells were trypsinized, and tumor sphere formation was performed as described above. Alternatively, the percentage of CD133^+^ cells of the trypsinized cells was analyzed by flow cytometry.

### In vivo tumorigenicity of lung cancer cells

Severe combined immunodeficient (SCID) mice (20 g, 6–8 weeks) provided by the Animal Breeding Center in Shanghai Institutes for Biological Sciences (Shanghai, China) were bred in specific pathogen-free (SPF) conditions. All procedures were approved by the Committee on Animal of the Second Military Medical University (Shanghai, China) and executed in accordance with guidelines of the Committee on Animal of the Second Military Medical University. The in vivo tumorigenicity assay was performed in SCID mice as described below. Briefly, lung cancer cells (2 × 105) were blended with BD Matrigel™ and implanted subcutaneously into SCID mice. CD133^+^ and CD133^−^ lung cancer cells were inoculated in both flanks of the same mice. After inoculation, the tumor formation was monitored in 50 days. Tumor volume = (width^2^ × length)/2.

### Statistical analysis

The statistic package SPSS 13.0 (SPSS Inc., Chicago, IL) was taken to analyze the data. A direct comparison between two groups was conducted with a Student’s non-paired *t* test. One-way analysis of variance (ANOVA) with the Dunnett’s or Newman–Keuls post-test compared the means of three or more groups. *P* value of < 0.05 was considered statistically significant.

## Results

### The characteristics of nanomicelles

**Fig. 1:**
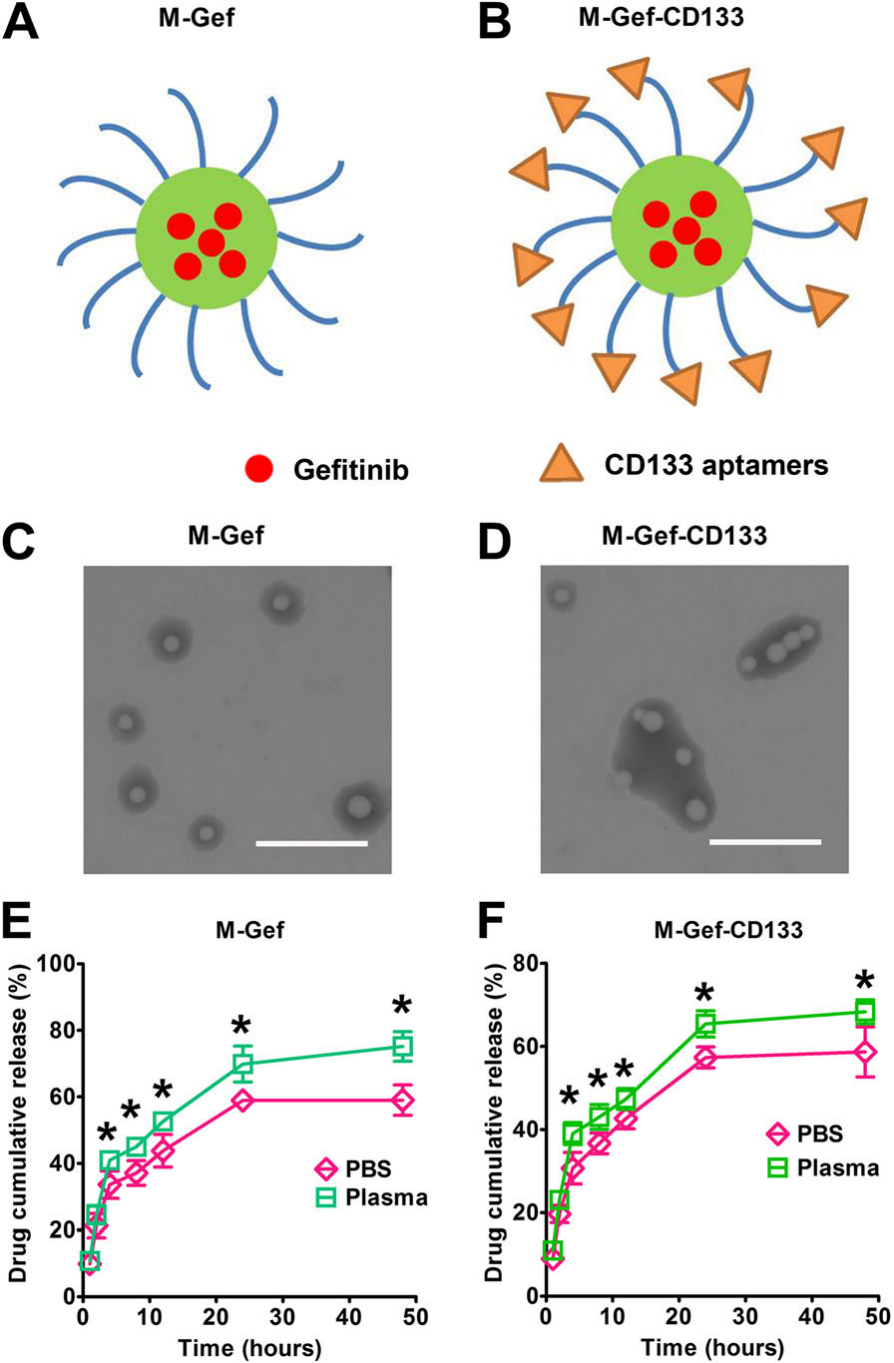
Characteristics of nanomicelles. **a**, **b** Schematic representations of M-Gef and M-Gef-CD133. **c**, **d** Transmission electron microscopy (TEM) revealed the morphology of the nanomicelles. The bars represent 100 nm. **e**, **f** The cumulative gefitinib release from nanomicelles in PBS and PBS with 10% plasma, respectively. Student’s non-paired *t* tests were taken to compare the two groups at different time points. Data are expressed as mean ± SD (*n* = 3)

The nanomicelles were fabricated by a lipid-film approach, and CD133 aptamers were conjugated to the nanomicelles by a simple thiol-maleimide reaction (Fig. 1a). As described in Table 1, all nanomicelles were rather small (~ 20 nm diameter), with a small polydispersity (PDI) (< 0.2) indicating the narrow and homogenous size distribution. The high negative zeta potential (about − 20 mV) of our prepared nanomicelles predicated high stability in circulation. The EE of the nanomicelles was 8~9%, and their drug-loading efficiency was > 85%, suggesting that lipid film is effective in the encapsulation of gefitinib in nanomicelles. Thus, our prepared nanomicelles possess appropriate size, zeta potential, and drug-loading capacity.

**Table 1:** Characteristics of nanomicelles

Nanomicelles	Size (nm)	PDI	Zeta potential (mV)	EE (%)	Drug loading (%)
M-Gef	21.3 ± 3.5	0.13 ± 0.04	− 16.8 ± 4.7	86.8 ± 9.2	9.1 ± 3.1
M-Gef-CD133	25.8 ± 4.4	0.17 ± 0.06	− 18.1 ± 4.1	85.9 ± 3.7	8.3 ± 5.8
M-CD133	20.9 ± 5.8	0.16 ± 0.05	− 19.2 ± 3.8	–	–

*PDI* polydispersity, *EE* encapsulation efficiency

Data are expressed as mean ± SD (*n* = 3)

As shown in Fig. 1c, d, both nanomicelles showed spherical shape and a disseminated pattern. M-Gef-CD133 had aggregate property which is distinct from M-Gef, which may be attributed to intermolecular forces of the aptamers conjugated to M-Gef-CD133. The in vitro release of gefitinib from M-Gef and M-Gef-CD133 was investigated (Fig. 1e, f). Both nanomicelles showed faster release in PBS with 10% plasma compared with PBS. For M-Gef and M-Gef-CD133, the drug release in PBS with 10% plasma was faster than that in PBS after 4 h (*P* < 0.05). A fast release of gefitinib (~ 50%) in the initial 12 h was observed. In the next 36 h, the cumulated gefitinib release of the nanomicelles was 60~70%. Taken together, both nanomicelles showed sustained drug release during 48 h.

### CD133^+^ population of lung cancer cells exhibits the features of CSCs

**Fig. 2:**
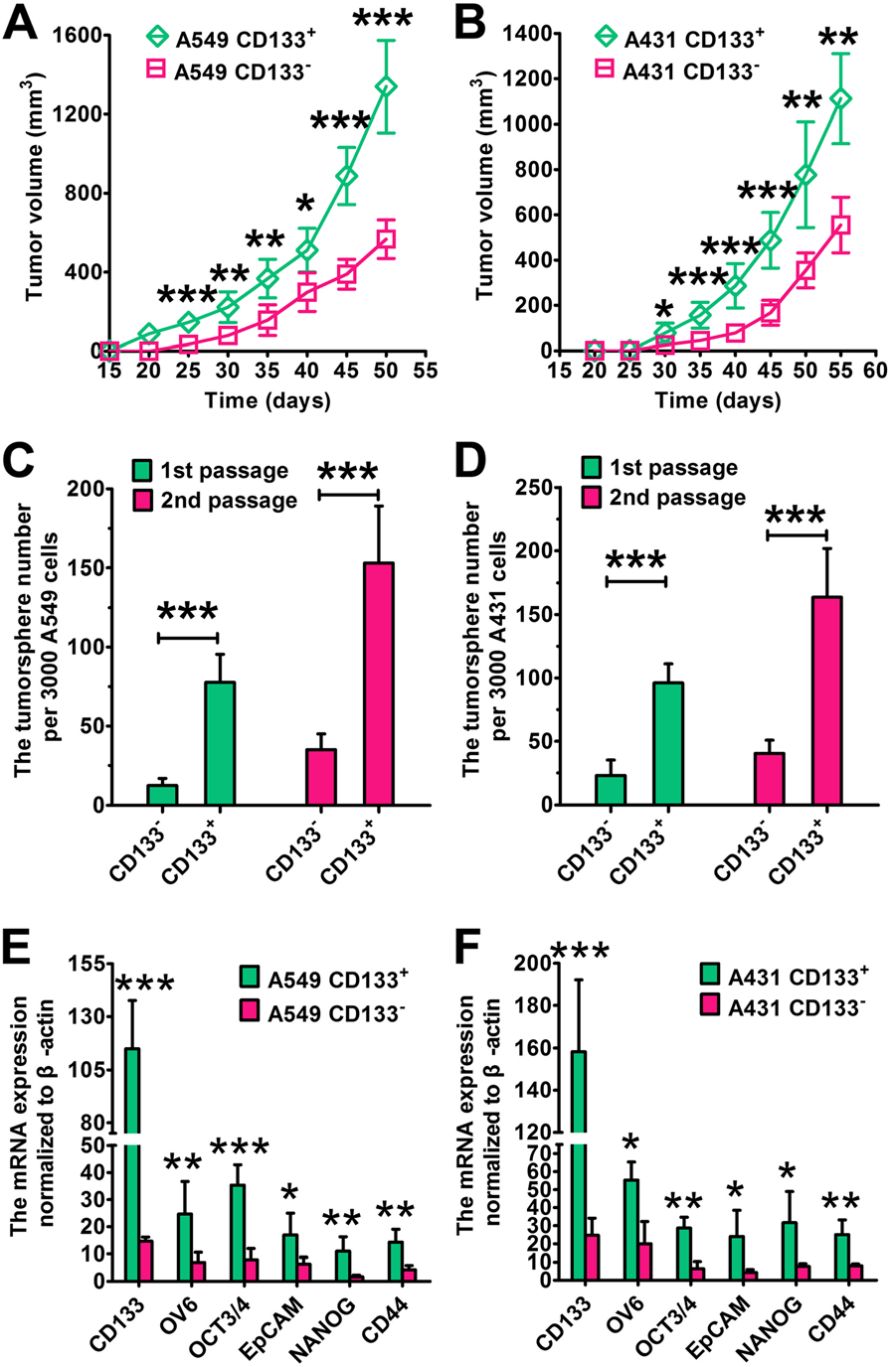
CD133^+^ lung cancer cells possess the characteristics of lung CSCs. **a**, **b** Tumor growth curve. CD133^+^ or CD133^−^ lung cancer cells (2 × 10^5^) were implanted subcutaneously into SCID mice. The tumor volume = (width^2^ × length)/2. Data are expressed as mean ± SD (*n* = 6). **c**, **d** CD133^+^ lung cancer cells produced more tumor spheres compared with CD133^−^ lung cancer cells. Data are expressed as mean ± SD (*n* = 3). **e**, **f** The mRNA level normalized to β-actin analyzed by RT-PCR. Data are expressed as mean ± SD (*n* = 3). Student’s non-paired *t* tests were taken to compare the two groups

After magnetic cell sorting, the percentage of CD133^+^ cells in lung cancer cells was > 98%. In contrast, the percentage of CD133^+^ cells was ~ 5% in the original lung cancer cell lines. CD133^+^ cells were more efficient in xenograft tumor formation than CD133^−^ cells (Fig. 2a, b). The average tumor volume of CD133^+^ cells was significantly larger compared with CD133^−^ cells after day 25 for A549 and day 30 for A431 (*P* < 0.05). The average tumor volume of CD133^+^ A549 cells was 1340 mm^3^ on day 50, significantly larger than that of CD133^−^ A549 cells (567 mm^3^, *P* < 0.001) (Fig. 2a). In A431 cells, similar results were obtained (Fig. 2b). Sphere-forming assays are utilized to identify stem cells on the account of their capacity of self-renewal and differentiation at the levels of single cells [11, 12, 29]. CD133^+^ A549 cells generated more tumor spheres than CD133^−^ A549 cells (first-passage: 77 vs. 12 for CD133^+^ vs. CD133^−^, *P* < 0.001; second-passage: 153 vs. 35 for CD133^+^ vs. CD133^−^, *P* < 0.001) (Fig. 2c). In the case of A431 cells, similar results were obtained (first-passage: 96 vs. 23 for CD133^+^ vs. CD133^−^, *P* < 0.001; second-passage: 163 vs. 40 for CD133^+^ vs. CD133^−^, *P* < 0.001) (Fig. 2d). The CSCs genes in both CD133^+^ and CD133^−^ cells was measured (Fig. 2e and f). The mRNA level of CD133 was increased by 10-fold in A549 CD133^+^ cells than A549 CD133^−^ cells. The mRNA levels of OV6, OCT3/4, EpCAM, NANOG, and CD44 were also significantly higher in A549 CD133^+^ cells than A549 CD133^−^ cells (*P* < 0.05). In the case of A431 cells, similar results were obtained. Taken together, CD133^+^ lung cancer cells showed the characteristics of lung CSCs.

### In vitro cellular uptake of nanoparticles

**Fig. 3:**
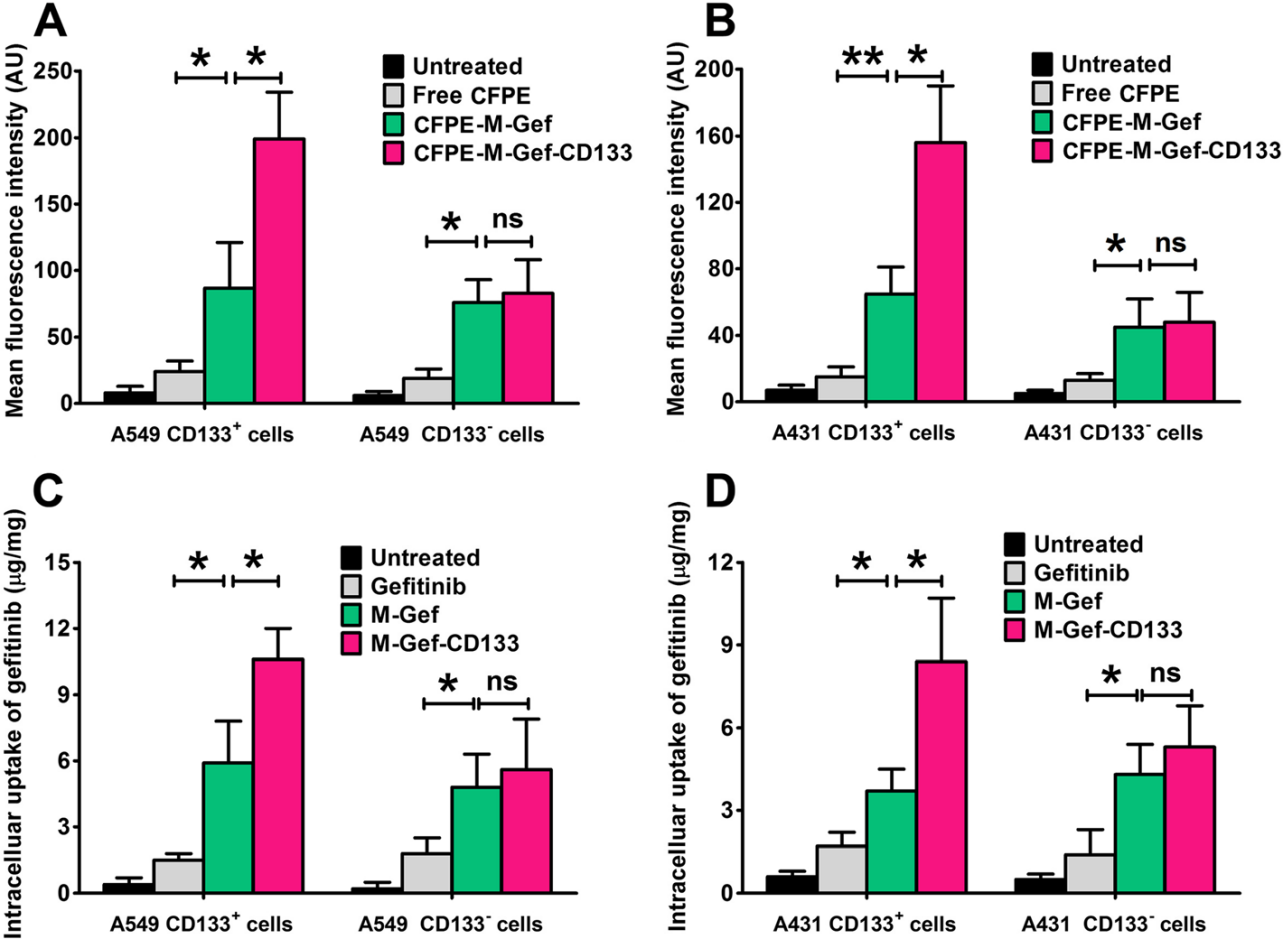
In vitro cellular uptake of nanomicelles. **a**, **b** The evaluation of in vitro cellular uptake of CFPE-labeled nanomicelles by flow cytometry. **c**, **d** In vitro cellular uptake of nanomicelles evaluated by HPLC. The intracellular uptake of gefitinib = intracellular uptake of gefitinib concentration/intracellular protein concentration × 100%. One-way ANOVA with the Newman–Keuls post-test was taken to compare the two groups. Data are expressed as mean ± SD (*n* = 3)

As shown in Fig. 3a, b, in A549 CD133^+^ cells, the mean fluorescence intensity in the CFPE-M-Gef-CD133-treated group was significantly higher compared with CFPE-M-Gef (*P* < 0.05) and CFPE-treated (*P* < 0.05). However, we did not observe any significant difference in mean fluorescence intensity between CFPE-M-Gef-CD133 and CFPE-M-Gef-treated groups in A549 CD133^−^ cells (*P* > 0.05). In the case of A431 cells, similar results were obtained (Fig. 3b). Next, we examined the quality of internalized gefitinib (Fig. 3c, d). The gefitinib concentration in the M-Gef-CD133-treated group was significantly higher compared with that in the M-Gef (*P* < 0.05) and gefitinib-treated groups (*P* < 0.05) in A549 CD133^+^ cells, whereas we did not observe any significant difference in gefitinib concentration between the M-Gef-CD133 and M-Gef-treated groups in A549 CD133^−^ cells (*P* > 0.05). In the case of A431 cells, similar results were obtained (Fig. 3d). Taken together, these results confirm that M-Gef-CD133 showed significantly increased targeting efficacy toward CD133^+^ lung cancer cells compared with M-Gef.

### CCK-8 assays

**Fig. 4:**
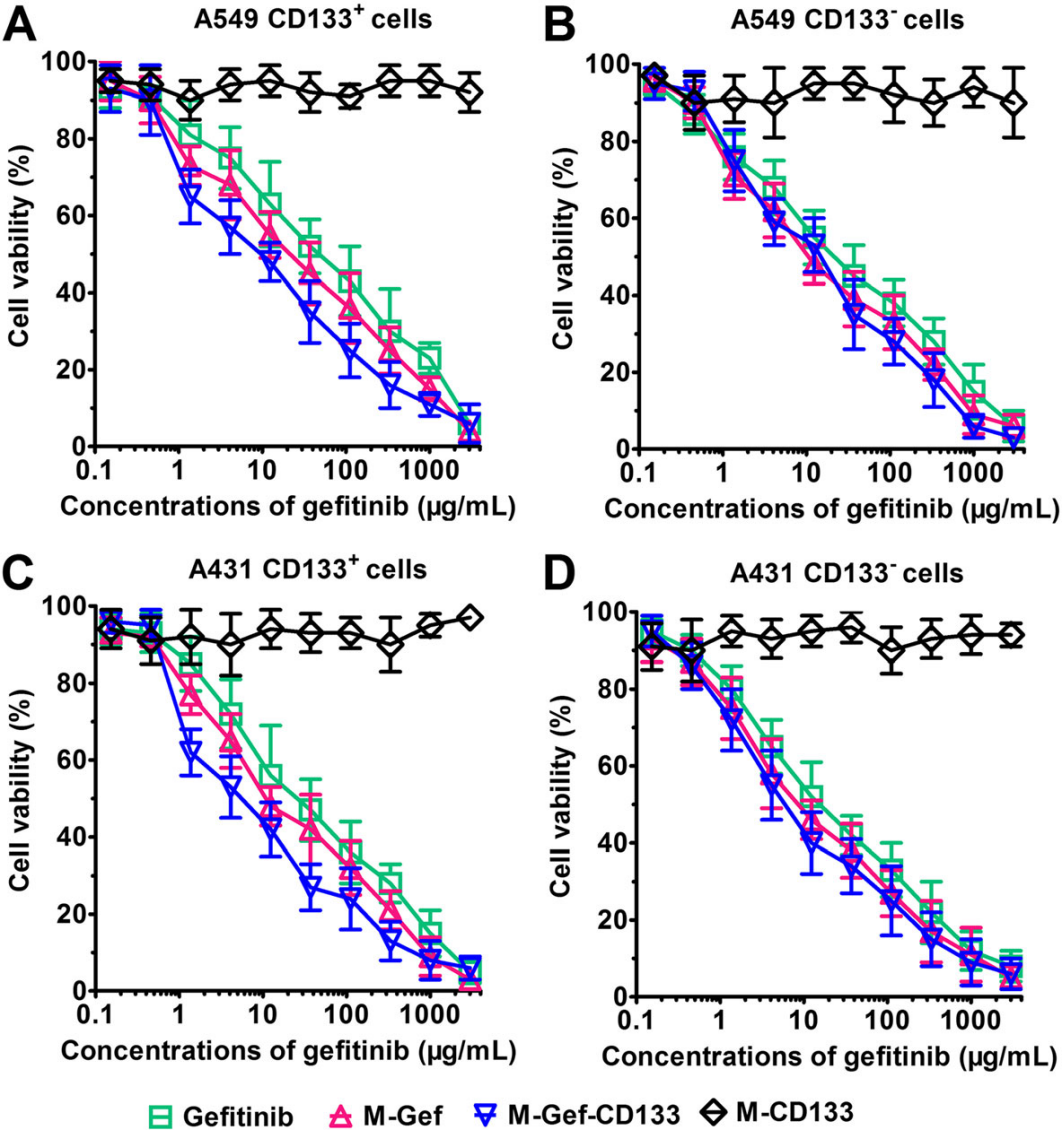
Cell proliferation assay evaluated using the CCK-8 kit. **a** A549 CD133^+^. **b** A549 CD133^−^. **c** A431 CD133^+^. **d** A431 CD133^−^. Data are expressed as mean ± SD (*n* = 3)

Figure 4 showed that M-CD133, blank nanomicelles with CD133 aptamers, did not induce potent cytotoxicity. In contrast, gefitinib, M-Gef, and M-Gef-CD133 showed dose-dependent cytotoxicity toward CD133^+^ and CD133^−^ lung cancer cells. We used IC_50_ values to quantitatively measure the in vitro cytotoxicity (Table 2). The IC_50_ value of M-Gef-CD133 (8.5 μg/mL) was significantly lower compared with M-Gef (31.9 μg/mL) (*P* < 0.01) and gefitinib (78.6 μg/mL) (*P* < 0.001) in A549 CD133^+^ cells. On the contrary, the IC_50_ value of M-Gef-CD133 (19.8 μg/mL) did not differ significantly compared with M-Gef (21.5 μg/mL) and gefitinib (31.3 μg/mL) in A549 CD133^−^ cells (*P* > 0.05). Thus, M-Gef-CD133 was 3.8- or 9.2-fold more effective than M-Gef or gefitinib in A549 CD133^+^ cells, respectively. Similar results were obtained in A431 CD133^+^ and CD133^−^ cells, where M-Gef-CD133 was 3.7- or 7.6-fold more effective than M-Gef or gefitinib in A431 CD133^+^ cells, respectively (*P* < 0.05). Thus, the enhanced cytotoxic effect of M-Gef-CD133 in CD133^+^ lung cancer cells could be due to their high CD133 expression.

**Table 2:** Cell proliferation assay of nanomicelles or free drugs at 48 h^a^

IC_50_ (μg/mL)	A549		A431	
	CD133^+^	CD133^−^	CD133^+^	CD133^−^
Gefitinib	78.6 ± 22.5	31.3 ± 13.5	35.8 ± 17.3	17.9 ± 8.4
M-Gef	31.9 ± 11.1	21.5 ± 13.6	17.6 ± 9.9	11.6 ± 9.5
M-Gef-CD133	8.5 ± 5.7	19.8 ± 8.4	4.7 ± 3.2	13.3 ± 5.6
M-CD133	> 3000	> 3000	> 3000	> 3000

^a^Data are expressed as mean ± SD (*n* = 3). The items are defined as follows: M-Gef indicates gefitinib-loaded nanomicelles, M-Gef-CD133 indicates gefitinib-loaded nanomicelles conjugated with CD133 aptamers, and M-CD133 indicates blank nanomicelles conjugated with CD133 aptamers

### Effect of nanomicelles on the CSC proportion in lung cancer cells

**Fig. 5:**
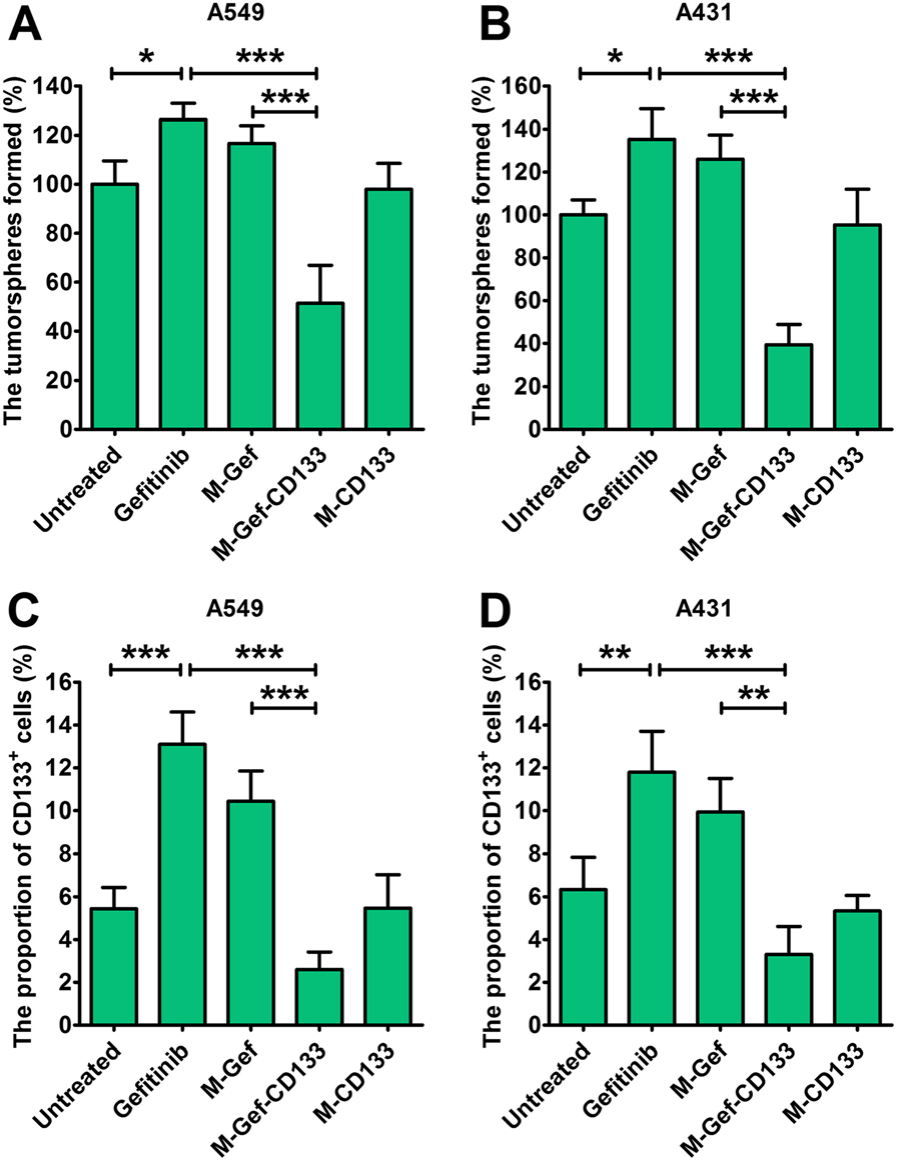
The evaluation of effect of nanomicelles or free drugs on the percentage of CSCs in lung cancer cells by tumor sphere formation assays (**a**, **b**) and percentages of CD133^+^ cells (**c**, **d**). The tumor sphere formation and percentages of CD133^+^ cells were examined after the treatment of A549 and A431 cells. The tumor sphere formation rate of the untreated group is used as a control (the rate is defined as 100%). One-way ANOVA with the Newman–Keuls post-test was taken to compare the two groups. Data are expressed as mean ± SD (*n* = 3)

The tumor sphere formation examined the effect of nanomicelles on CSCs percentage (Fig. 5). Strikingly, in A549 cells, gefitinib and M-Gef treatment significantly increased the number of tumor spheres (*P* < 0.05), suggesting that gefitinib and M-Gef may increase the percentage of CSCs in lung cancer after treatment (Fig. 5a). Notably, relative to that in the untreated control, M-Gef-CD133 induced a twofold decrease in the number of A549 tumor spheres. The number of tumor spheres after M-Gef-CD133 treatment was smaller than gefitinib (*P* < 0.001) and M-Gef (*P* < 0.001) treatment in A549 cells. Consistently, the percentage of CD133^+^ A549 cells was significantly decreased after M-Gef-CD133 treatment than gefitinib (*P* < 0.001) and M-Gef (*P* < 0.001) treatment (Fig. 5c).

Similarly, relative to that in the untreated control, M-Gef-CD133 induced a 2.5-fold decrease in the number of A431 tumor spheres (Fig. 5b). The number of tumor spheres after M-Gef-CD133 treatment is smaller compared with gefitinib (*P* < 0.001) and M-Gef (*P* < 0.001), although the number of tumor spheres was significantly increased after the treatment with gefitinib and M-Gef (*P* < 0.05). Consistently, M-Gef-CD133 treatment significantly decreased the percentage of CD133^+^ A431 cells than gefitinib (*P* < 0.001) and M-Gef (*P* < 0.01) treatment (Fig. 5d).

Taken together, M-Gef-CD133 was demonstrated to decrease the CSC proportion in lung cancer cells, whereas gefitinib and M-Gef only increased the proportion.

## Discussion

Owing to the high expression of CD133 in lung CSCs, we utilized the interaction of CD133 aptamers and CD133 as a promising approach to realize effective delivery of gefitinib to CD133-overexpressing lung CSCs. M-Gef-CD133 was developed and demonstrated to possess the potential to eliminate CD133^+^ lung CSCs.

Gefitinib was the first approved tyrosine kinase inhibitors (TKIs) for NSCLC [30]. However, resistance to gefitinib limits progression-free survival among patients with NSCLC [31]. The EGFR T790M secondary mutation is a well-known mechanism of gefitinib resistance [29]. Recently, studies have demonstrated that lung CSCs are involved as nonmutational mechanisms conferring resistance to gefitinib in NSCLC [5, 32, 33]. Consistent with those previous studies, gefitinib was demonstrated to show significantly reduced cytotoxic effects in CD133^+^ lung CSCs and increased the tumor sphere formation and proportion of CD133^+^ lung CSCs, suggesting that lung CSCs are resistant to gefitinib. In contrast, M-Gef-CD133 showed much higher toxicity toward CD133^+^ lung CSCs than toward CD133^−^ lung cancer cells, indicating that M-Gef-CD133 overcame the gefitinib resistance. To the best of our knowledge, this is the first research that utilizes nanoparticles to overcome the gefitinib resistance of lung CSCs. As stated by Gao et al., nanoparticles represent a promising approach in eradicating CSCs by several potential mechanisms, including by improving the unfavorable pharmaceutical properties of drugs, utilizing CSC phenotype-specific ligands, and bypassing or inhibiting the efflux pump on CSCs [34].

The small size of our prepared nanomicelles (~ 20 nm) may be beneficial for their targeting efficacy for lung CSCs, since the size of nanoparticles is critical for their tumor penetration [35–37]. Cabral reported that the penetration ability of nanoparticles smaller than 50 nm was better than those larger than 50 nm [38]. Considering that high concentration of CSCs are often found in the central necrotic and hypoxic regions of solid tumors [39, 40], it is critical that nanoparticles possess superior penetration into solid tumors to kill CSCs. Thus, our prepared nanomicelles are expected to have superior penetration in solid tumors and kill CSCs efficiently. Owing to a technical limitation, we could not perform the penetration assay of our prepared M-Gef-CD133 in a xenograft mouse model. However, we fully realize the importance of testing the penetration of M-Gef-CD133 and will perform that assay in the future when the conditions permit.

Although gefitinib shows potent activity toward lung cancer, its solubility in water is poor [41]. Nanoparticles show promise to improve the water solubility of chemotherapy drugs [7]. One example is Abraxane® which could significantly increase the drug concentrations in cancer [42]. In our study, we used nanomicelles made of DSPE-PEG2000—commercially available FDA-approved biodegradable lipid polymers—as the drug delivery system to increase gefitinib’s solubility and its targeting toward lung CSCs. Further, CD133 aptamers was pivotal in guaranteeing the targeting efficacy of M-Gef-CD133 to CD133^+^ lung cancer cells. First, we used flow cytometry and HPLC to demonstrate that M-Gef-CD133 could efficiently bind to CD133^+^ lung CSCs. Upon cell binding, M-Gef-CD133 induced enhanced cytotoxicity compared with non-targeted M-Gef and gefitinib in CD133^+^ lung CSCs, but not in CD133^−^ lung cancer cells. We observed that M-Gef-CD133 reduced the number of tumor spheres and the percentage of CD133^+^ lung CSCs more effectively than M-Gef and gefitinib, suggesting that M-Gef-CD133 preferably eliminates CD133^+^ lung CSCs. In contrast, M-Gef and gefitinib increased the number of tumor spheres and the percentage of CD133^+^ lung CSCs. Taken together, our data have demonstrated that M-Gef-CD133 could promote the selective toxicity of gefitinib against CD133^+^ lung cancer cells.

There is one limitation in our study. It is needed that the lung cancer cell lines with EGFR mutations which are sensitive to gefitinib are tested in this study. However, the two available lung cancer cell lines with EGFR mutations in our lab could not form tumor spheres and thus could not be tested in this study. We completely recognize the importance of testing our nanoparticles in lung cancer cell lines with EGFR mutations and will do that in the future if possible.

## Conclusion

This is the first study that utilizes nanoparticles to overcome the gefitinib resistance of lung CSCs. Our data demonstrated that CD133 is a promising target for the specific delivery of gefitinib to lung CSCs. M-Gef-CD133 could efficiently deliver gefitinib to CD133^+^ lung CSCs and induce selective toxicity against CD133^+^ lung CSCs. Taken together, M-Gef-CD133 represents a promising approach to target lung CSCs.

## Abbreviations

DSPE-PEG2000 (ammonium salt): 1,2-Distearoyl-sn-glycero-3-phosphoethanolamine-*N*-[methoxy(polyethylene glycol)-2000]
DSPE-PEG2000-Mal (ammonium salt): 1,2-Distearoyl-sn-glycero-3-phosphoethanolamine-*N*-[maleimide(polyethylene glycol)-2000]
CFPE (ammonium salt): 1,2-Dioleoyl-sn-glycero-3-phosphoethanolamine-*N*-carboxyfluorescein
ANOVA: Analysis of variance
ATCC: American Type Culture Collection
bFGF: Basic fibroblast growth factor
CD133: Cluster of differentiation 133
cDNA: Complementary DNA
CSCs: Cancer stem cells
EDC: 1-Ethyl-3-3-dimethylaminopropyl carbodiimide
EE: Encapsulation efficacy
EGF: Epidermal growth factor
EGFR: Epidermal growth factor receptor
FBS: Fetal bovine serum
FDA: Food and Drug Administration
HPLC: High performance liquid chromatography
ITS: Insulin-transferrin-selenium
M-Gef: Gefitinib-loaded nanomicelles
M-Gef-CD133: Gefitinib-loaded poly(ethylene glycol) 2000-distearoylphosphatidylethanolamine nanomicelles conjugated with CD133 aptamers
NHS: *N*-Hydroxysuccinimide
NSCLC: Non-small cell lung cancer
PDI: Polydispersity
PE: Phycoerythrin
RT-PCR: Real-time polymerase chain reaction
TEM: Transmission electron microscopy
TKIs: Tyrosine kinase inhibitors
